# Structural and Optoelectronic Properties of Two-Dimensional Ruddlesden–Popper Hybrid Perovskite CsSnBr_3_

**DOI:** 10.3390/nano11082119

**Published:** 2021-08-20

**Authors:** Guangbiao Xiang, Yanwen Wu, Yushuang Li, Chen Cheng, Jiancai Leng, Hong Ma

**Affiliations:** 1 Shandong Provincial Key Laboratory of Optics, Photonic Device and Collaborative Innovation Center of Light Manipulations and Applications, School of Physics and Electronics, Shandong Normal University, Jinan 250014, China; m17753643157@163.com (G.X.); ywwu_1209@163.com (Y.W.); lys3573172070@163.com (Y.L.); 2 School of Electronic and Information Engineering (Department of Physics), Qilu University of Technology (Shandong Academy of Sciences), Jinan 250353, China

**Keywords:** 2D Ruddlesden–Popper hybrid perovskites, first-principles study, band structures, optoelectronic properties

## Abstract

Ultrathin inorganic halogenated perovskites have attracted attention owing to their excellent photoelectric properties. In this work, we designed two types of Ruddlesden–Popper hybrid perovskites, Cs*_n_*_+1_Sn*_n_*Br_3*n*+1_ and Cs*_n_*Sn*_n_*_+1_Br_3*n*+2_, and studied their band structures and band gaps as a function of the number of layers (*n* = 1–5). The calculation results show that Cs*_n+_*_1_Sn*_n_*Br_3*n*+1_ has a direct bandgap while the bandgap of Cs*_n_*Sn*_n_*_+1_Br_3*n*+2_ can be altered from indirect to direct, induced by the 5*p*-Sn state. As the layers increased from 1 to 5, the bandgap energies of Cs*_n_*_+1_Sn*_n_*Br_3*n*+1_ and Cs*_n_*Sn*_n_*_+1_Br_3*n*+2_ decreased from 1.209 to 0.797 eV and 1.310 to 1.013 eV, respectively. In addition, the optical absorption of Cs*_n_*_+1_Sn*_n_*Br_3*n*+1_ and Cs*_n_*Sn*_n_*_+1_Br_3*n*+2_ was blue-shifted as the structure changed from bulk to nanolayer. Compared with that of Cs*_n+_*_1_Sn_n_Br_3*n+*1_, the optical absorption of Cs*_n_*Sn*_n_*_+1_Br_3*n*+2_ was sensitive to the layers along the *z* direction, which exhibited anisotropy induced by the SnBr_2_-terminated surface.

## 1. Introduction

Perovskites have become competitive candidate materials for photovoltaic and optoelectronic applications, such as solar cells, optically pumped lasers, detectors and light emitting diodes [1,2,3,4,5]. At present, the power-conversion efficiency of perovskite solar cells has reached 25.4% [6]. Over the past several years, the high-performance perovskite light-emitting diodes have developed rapidly, reaching high external quantum efficiencies of over 20% [1]. At room temperature, the optically pumped laser made from the lead halide perovskite nanowires can be tuned in the whole visible spectrum region (420−710 nm) with high-quality factors and low lasing thresholds [3]. The advantages of these materials include their long carrier lifetimes and diffusion lengths, large absorption coefficients in the visible spectrum, small effective masses, tunable bandgaps and high quantum efficiency [7,8,9,10,11,12]. In addition, compared with other materials, perovskites have the advantage of low-cost and facile processing [13,14]. Although many achievements in the application of lead-based perovskites have been obtained, there are still many challenges, especially due to the presence of toxic lead. The toxicity of lead can cause serious damage to life and the environment. Therefore, the use of less toxic materials, such as tin and other alkaline earth metals, to replace lead has been widely investigated [15]. An inorganic substitute can reduce hysteresis loss that is caused by the presence of methylammonium. Previous work showed that CsPbBr_3_ solar cells are as efficient as and have higher levels of environmental stability than CH_3_NH_3_PbBr_3_ solar cells, even after aging for 2 weeks [16].

Recently, two-dimensional (2D) Ruddlesden–Popper (RP) hybrid perovskite materials have attracted intensive attention because of their wide applications [17,18,19,20,21,22,23]. Compared with bulk perovskites, 2D perovskites show many unique properties. For example, 2D RP perovskites have a narrow full width at half maximum and blue-shift photoluminescence signal with the decrease in the number of layers owing to the quantum confinement of carriers [24]. Two-dimensional RP perovskites have a higher chemical stability because of the linking of organic molecules, such as CH_3_(CH_2_)_3_NH_3_ and CH_3_NH_3_, on the terminated surface [25]. Ultrathin 2D RP perovskite solar cells can also reduce production costs from the point of view of mass production [26,27]. Until recently, the layer numbers and the size of synthesized ultrathin 2D RP films have been accurately controlled experimentally. Importantly, the bandgap of 2D RP perovskites can be easily adjusted by changing the number of layers [28]. Therefore, the layer-dependent properties of perovskites further enrich their applications in optoelectronic and photovoltaic fields [28,29,30]. In addition, it is generally known that different terminated surfaces of 2D RP perovskites are important for optoelectronic properties and applications, such as chemical activity of the surfaces, sensing effects, and ultrathin film production. Previous work has reported that the iodine defect improves the efficiency of photoabsorbance in a SnBr_2_-terminated surface perovskite solar cell by lowering its energy gap [31]. Ab initio calculations of the structural and electronic properties of the CsBr- and CaBr_2_/GeBr_2_/SnBr_2_-terminated (001) surfaces of CsMBr_3_ (M = Ca, Ge, Sn) perovskites indicated noticeable changes of the surface properties in comparison with those for bulk materials [32]. Thus far, there have been many literatures about 2D perovskites with different surface terminations, including the structural and electronic properties, surface relaxations, energetics, bonding properties, ferroelectrics and dipole moment [33,34,35,36,37,38,39,40]. The SrTiO_3_ (100) surface relaxation and rumpling have been calculated with two different terminations (SrO and TiO_2_) [39]. Experimentally, the researchers have already studied the ferroelectric relaxation, surface relaxation, polar oxide and surface rumpling of perovskites with different surface terminations [41,42,43,44,45,46]. Particularly, A. Ikeda et al. determined surface rumpling and relaxation of TiO_2_-terminated SrTiO_3_ (001) using the medium energy ion scattering, and found that the occupation fraction of the TiO_2_ face ranged from 85 to 95% [45].

Herein, we have designed two models based on CsSnBr_3_ with different surface terminations: Cs*_n_*_+1_Sn*_n_*Br_3_*_n_*_+1_ and Cs*_n_*Sn*_n_*_+1_Br_3_*_n_*_+2_. The terminated surfaces are CsBr and SnBr_2_ for the two models, respectively. We investigated the structural and optoelectronic properties, including band structures, surface relaxation effects, density of states and optical absorption spectra of the two models using the first-principles method. Cs*_n_*_+1_Sn*_n_*Br_3_*_n_*_+1_ and Cs*_n_*Sn*_n_*_+1_Br_3_*_n_*_+2_ with different numbers of layers showed different properties in the bandgap and absorption coefficient. The theoretical calculation results obtained in the present work can serve as a guideline in the design of different structures and for the improvement of the efficiency of optical absorbance for 2D inorganic perovskites.

## 2. Computational Model and Method

The first-principles calculation in this work is based on density functional theory (DFT) [47]. The calculation method was the all-electron-like projector augmented wave method and the exchange correlation potential realized by Perdew–Burke–Enserch (PBE) in the Vienna Ab Initio Simulation Package (VASP) [48,49,50]. The electron exchange correction function was described by the generalized gradient approximation parameterized by PBE. The cut-off energy of the plane wave was set to 500 eV. All atoms were allowed to relax until the Hellmann–Feynman forces reached the convergence criterion of less than 0.01 eV/Å. The convergence threshold of energy was set at 10^−5^ eV. The Monkhorst-Pack scheme was used to sample *k*-points in the Brillouin zone [51]. The *k*-point meshes were set to 6 × 6 × 1 and 8 × 8 × 1 for the electronic structure and density of states, respectively. The HSE06 hybrid functional used to calculate the bandgap and the fraction of exact exchange in the Hartree–Fock/DFT hybrid functional-type calculation was 25%. The spin–orbital coupling (SOC) interaction of the Sn atom is weaker than that of heavy atom lead, so the SOC interaction is ignored in our calculation.

**Figure 1:**
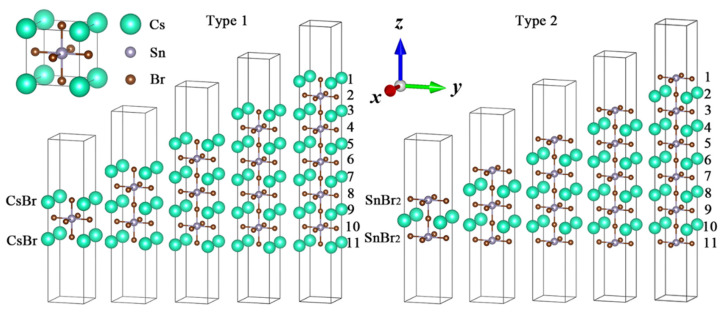
Surface models of ultrathin CsSnBr_3_ perovskites: (**left**) CsBr termination for Type 1 and (**right**) SnBr_2_ termination for Type 2. The top left corner is a model of bulk CsSnBr_3_.

As mentioned earlier, a differently terminated surface will change the band structure, optical absorption and bandgap energy of perovskites. In order to further explore the layer dependence of different surface terminations, we designed two models, namely, Cs*_n_*_+1_Sn*_n_*Br_3_*_n_*_+1_ and Cs*_n_*Sn*_n_*_+1_Br_3_*_n_*_+2_ (*n* = 1–5), as Type 1 and Type 2 based on the cubic phase structure (Pm3m space group) of three-dimensional CsSnBr_3_ [52], shown in Figure 1. A two-layer Type 2 molecule (Cs_2_Sn_3_Br_8_) was structured by a one-layer Type 1 molecule (Cs_2_Sn_1_Br_4_), which added one plane composed by one Sn atom and four Br atoms on both the top and bottom surfaces, respectively. The same rule can be applied to multiple-layer structures, namely Cs*_n_*Sn*_n_*_+1_Br_3_*_n_*_+2_ (*n* = *j +* 1), which contain Cs*_n_*_+1_Sn*_n_*Br_3_*_n_*_+1_ (*n* = *j*). A vacuum region of 10 Å in the *z* direction was set on the bottom and top of the models to avoid interaction between the atoms. The electronic configurations of the chemical elements of Cs*_n_*_+1_Sn*_n_*Br_3_*_n_*_+1_ and Cs*_n_*Sn*_n_*_+1_Br_3_*_n_*_+2_ included 4*s*^2^4*p*^5^ (Br), 5*s*^2^5*p*^6^6*s*^1^ (Cs) and 5*s*^2^5*p*^2^ (Sn) [32].

## 3. Results and Discussion

In our simulation, the structures of Cs*_n_*_+1_Sn*_n_*Br_3_*_n_*_+1_ and Cs*_n_*Sn*_n_*_+1_Br_3_*_n_*_+2_ was optimized and all atoms were allowed to relax. The degree of surface rumpling was quantitatively described to reveal the difference between the two structures. A variable *d_i,i+_*_1_ can be defined as the interplanar distance between the neighboring atomic planes. The index *i* labels the atomic layers of Type 1 and Type 2 in Figure 1. The relative displacements are described using the following nondimensional quantity equation [32]:

(1) $$\delta_{i,i+1}=\frac{d_{i,i+1}-a_{0}/2}{a_{0}},$$

where *a*_0_ is the theoretical lattice constant calculated for bulk CsSnBr_3_.

*δ_i,i+_*_1_ is related with the vacuum layer. For example, in Cs_3_Sn_2_Br_7_ (*n* = 2) of Type 1, the relative displacements *δ*_1,2_ and *δ*_2,3_ were −1.05 and 1.69%, respectively. All relative displacements, *δ_i,i+_*_1_, are shown in Table 1 for layers 1 to 5. The structures of Cs*_n_*_+1_Sn*_n_*Br_3_*_n_*_+1_ and Cs*_n_*Sn*_n_*_+1_Br_3_*_n_*_+2_ were centrosymmetric and *δ_i,i+_*_1_ gradually decreased from the terminated surface to the symmetric center. This trend implies that the stability of the octahedral structure near the center of symmetry was better than that of the terminated surface. We also found that the relative displacements *δ*_1,2_ for Type 1 were bigger than that for Type 2, which means Cs*_n_*_+1_Sn*_n_*Br_3_*_n_*_+1_ with CsBr-termination had better stability than Cs*_n_*Sn*_n_*_+1_Br_3_*_n_*_+2_. Our conclusion is consistent with the previous calculation [32].

The ratio of angle change before and after optimization can be defined to correspond with the degree of surface rumpling with bond angles in the same atomic plane. The equation is as follows:

(2) $$\eta_{i,i+1}=\frac{\theta_{i,i+1}-\pi }{\pi },$$

where *θ* is the degree of Cs_i_Br_i_Cs_i_ or Br_i_Sn_i_Br_i_ (i = 1–5); namely, two Cs atoms are nonadjacent in the CsBr plane or two Br atoms are along the y direction in the SnBr_2_ plane. Compared with the angles of CsBrCs, the angles of BrSnBr forming octahedral frames change slightly. When *n* = 4 and 5, the layers rumpling, *η_i_*, of CsBrCs and BrSnBr decreased to zero from the terminated surface to the symmetric center. In Type 1, the structure of Cs*_n+_*_1_Sn_n_Br_3_*_n+_*_1_ contained one or more complete perovskite structures (ABX_3_), in which the shape of the band structure had no change and only the value of the band energy varied with the layers from 1 to 5. It is noticed that Cs_n_Sn*_n+_*_1_Br_3_*_n+_*_2_ contains SnBr_2_ termination on the top and bottom surfaces in addition to one or more ABX_3_ in the Type 1 model. Therefore, compared to the structures of Cs*_n+_*_1_Sn_n_Br_3_*_n+_*_1_ and Cs_n_Sn*_n+_*_1_Br_3_*_n+_*_2_, we inferred that the change of band structure shape of Cs_n_Sn*_n+_*_1_Br_3_*_n+_*_2_ was induced by the SnBr_2_-terminated surface.

**Figure 2:**
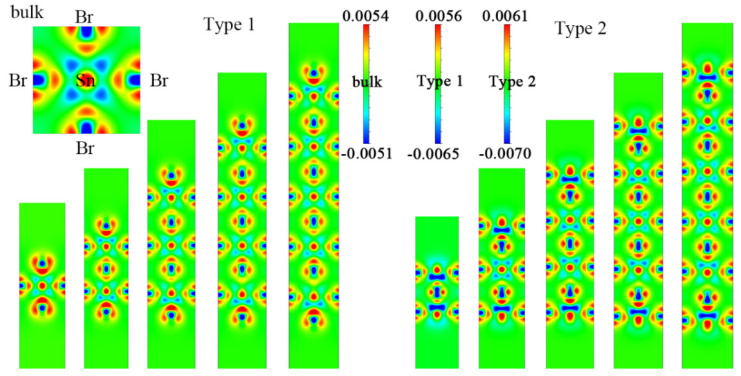
Electron density difference in the slice plane (0.5, 0, 0) in Cs*_n_*_+1_Sn*_n_*Br_3*n*+1_ and Cs*_n_*Sn*_n_*_+1_Br_3*n*+2_ (*n* = 1–5). The top left corner is the electron density difference of the bulk CsSnBr_3_. The scale is in electrons/Å.

Figure 2 shows the electron density difference of Cs*_n_*_+1_Sn*_n_*Br_3_*_n_*_+1_ and Cs*_n_*Sn*_n_*_+1_Br_3_*_n_*_+2_ in the slice plane (0.5, 0, 0) with different layer numbers. Obviously, the lost charge of the Sn atom transferred to the six adjacent Br atoms, and it is asymmetric along the *z* direction. The length of the Sn–Br bond is inversely proportional to the electron density; that is, the larger the electron density, the shorter the bond. This asymmetry gradually decreases along the *z* direction from the terminated surface to the symmetry center. This is consistent with the changes in *δ_i,i+_*_1_, which gradually decreased from the terminated surface to the center of symmetry.

**Figure 3:**
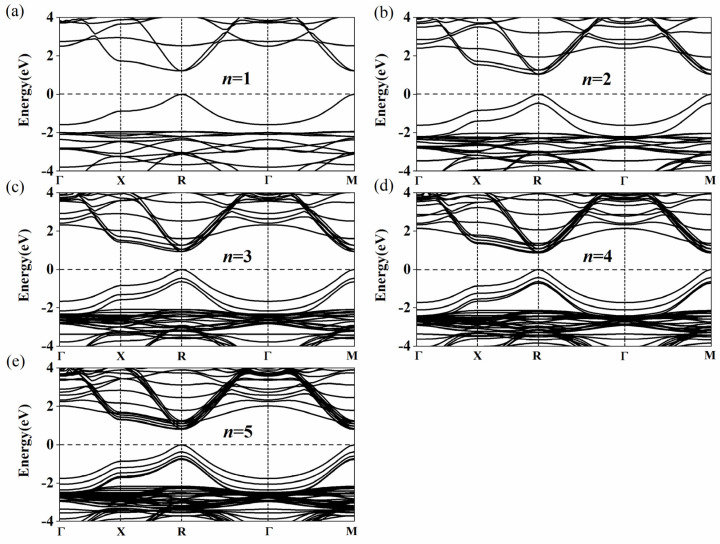
Calculated band structures of Cs*_n_*_+1_Sn*_n_*Br_3*n*+1_ for (**a**) 1 layer, (**b**) 2 layers, (**c**) 3 layers, (**d**) 4 layers and (**e**) 5 layers. The CBM and the VBM appear at the *R* point (0.5 0.5 0.5). The high symmetry path is assumed with respect to the Brillouin zone center *Γ* with the coordinates (0, 0, 0) to *X* (0, 0.5, 0), *R* (0.5, 0.5, 0.5), *Γ* (0, 0, 0) and *M* (0.5, 0.5, 0).

**Figure 4:**
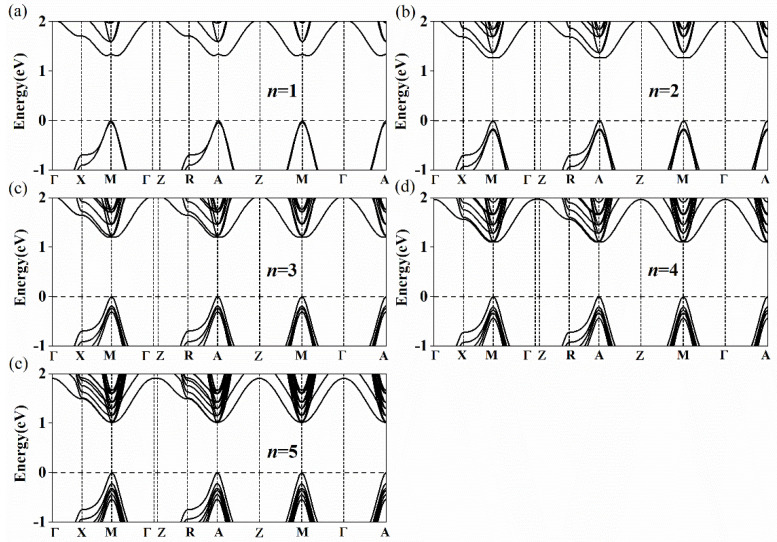
Calculated band structures of Cs*_n_*Sn*_n_*_+1_Br_3*n*+2_ for (**a**) 1 layer, (**b**) 2 layers, (**c**) 3 layers, (**d**) 4 layers and (**e**) 5 layers. The high symmetry path is assumed with respect to the Brillouin zone center *Γ* with the coordinates (0, 0, 0) to *X* (0, 0.5, 0), *M* (0.5, 0.5, 0), *Γ* (0, 0, 0), *Z* (0, 0, 0.5), *R* (0, 0.5, 0.5), *A* (0.5, 0.5, 0.5), *Z* (0, 0, 0.5), *M* (0.5, 0.5, 0), *Γ* (0, 0, 0) and *A* (0.5, 0.5, 0.5).

The calculated bandgap energies of Cs*_n+_*_1_Sn_n_Br_3_*_n+_*_1_ and Cs_n_Sn*_n+_*_1_Br_3_*_n+_*_2_ (*n* = 1–5) are listed in Table 2. As *n* increases from 1 to 5, the bandgap energies of Cs*_n+_*_1_Sn_n_Br_3_*_n+_*_1_ and Cs_n_Sn*_n+_*_1_Br_3_*_n+_*_2_ decrease from 1.209 to 0.797 eV and 1.310 to 1.013 eV, respectively. Our calculations are almost the same as those calculated by Anu et al. Their results showed that the bandgap energies of Cs*_n+_*_1_Sn_n_Br_3_*_n+_*_1_ are 1.2 eV, 1.04 eV, 0.92 eV, 0.85 eV and 0.79 eV with the layers from 1 to 5 [53]. In experiments, it was also found that the bandgap energies of (PEA)_2_(MA)*_n_*_–1_Pb*_n_*Br_3*n+*1_ (2D) and (CH_3_(CH_2_)_3_NH_3_)_2_(CH_3_NH_3_)*_n_*_−1_Pb_n_I_3*n+*1_ (2D) gradually shrink with the increase in the layer number [41,42]. There are many publications which support our models [31,32,54,55,56]. As shown in Figure 3a–e, the conduction band minimum (CBM) and the valence band maximum (VBM) of Type 1 appear at the R point (0.5, 0.5, 0.5). The bandgap of Cs*_n+_*_1_Sn_n_Br_3_*_n+_*_1_ presents a direct bandgap and gradually shrinks with the increase in the layer number. According to this trend, the bandgap of Cs*_n+_*_1_Sn_n_Br_3_*_n+_*_1_ will reach 0.641 eV with the increase of *n* [54]. The bandgap of Cs*_n_*Sn*_n+_*_1_Br_3_*_n+_*_2_ also decreases with the increase of *n*. However, unlike that of Cs*_n+_*_1_Sn_n_Br_3_*_n+_*_1_, the band structure of Cs_n_Sn*_n+_*_1_Br_3_*_n+_*_2_ in Figure 4 shows an indirect bandgap because the CBM of Cs_n_Sn*_n+_*_1_Br_3_*_n+_*_2_ does not appear at the M point (0.5, 0.5, 0) when *n* = 1, 2 and 3 (Figure 4a–c). It can be seen that the band structure at the bottom of the conduction band along the M→X and M→Γ directions is W-shaped (*n* = 1, 2 and 3) instead of parabolic, as shown in Figure 4. When *n* = 1, 2 and 3, the differences between the M point (0.5, 0.5, 0) and the lowest point are 26.6, 8.7 and 1.8 meV, respectively. With the increase of *n*, the differences gradually decrease to zero when *n* = 4 and 5 (Figure 4d,e), which means the band structure of Cs*_n_*Sn*_n+_*_1_Br_3_*_n+_*_2_ turns into a direct bandgap. The band structures of Cs*_n_*Sn*_n+_*_1_Br_3_*_n+_*_2_ (*n* = 1) calculated by PBE and HSE06 DFT are shown in Supplementary Figure S1. It is easy to see that the band structure of Cs*_n_*Sn*_n+_*_1_Br_3_*_n+_*_2_ (*n* = 1) calculated by HSE06 has a shift up compared with that by the PBE calculation, while the shape of the band does not change. In view of the high computational cost, PBE is used in this work.

**Figure 5:**
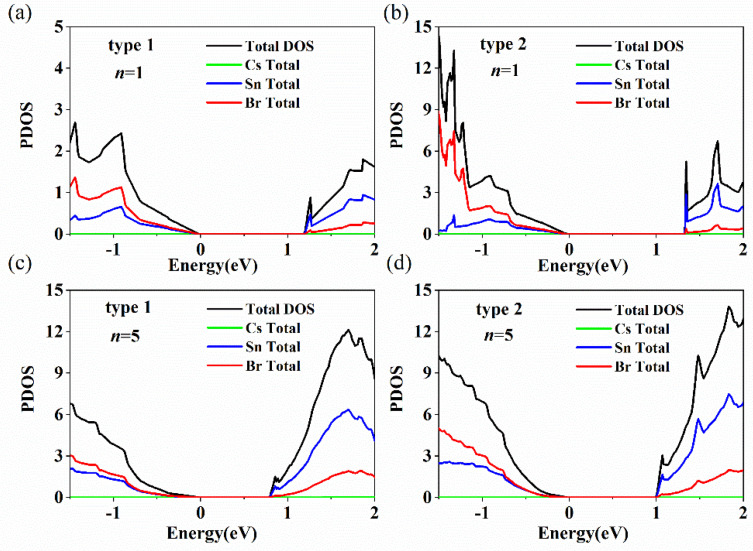
Calculated DOS for Cs*_n_*_+1_Sn*_n_*Br_3*n*+1_ and Cs*_n_*Sn*_n_*_+1_Br_3*n*+2_ with *n* = 1 and 5. The arrow points to the peaks of Type 1 and Type 2 at *n* = 1. The green, blue, red, and black lines represent Cs TDOS, Sn TDOS, Br TDOS and TDOS, respectively. The models are (**a**) Cs*_n_*_+1_Sn*_n_*Br_3*n*+1_ (*n* = 1), (**b**) Cs*_n_*Sn*_n_*_+1_Br_3*n*+2_ (*n* = 1), (**c**) Cs*_n_*_+1_Sn*_n_*Br_3*n*+1_ (*n* = 5) and (**d**) Cs*_n_*Sn*_n_*_+1_Br_3*n*+2_ (*n* = 5).

To carefully examine which atom induces the emergence of the indirect bandgap of Cs*_n_*Sn*_n+_*_1_Br_3_*_n+_*_2_, the density of states (DOS) of both types with *n* = 1 and 5 were calculated, as shown in Figure 5. Figure 5 indicates that the Sn state plays a dominant role, while the Cs state is negligible for the valence band top and conduction band bottom in both models. It can be seen that a small peak appears at the bottom of the conduction band of Cs*_n+_*_1_Sn*_n_*Br_3_*_n+_*_1_ (Type 1, *n* = 1; indicated by an arrow), which is induced by the degeneracy of energy levels (Figure 5a). Figure 5b shows that the valence band top and conduction band bottom of Cs*_n_*Sn*_n+_*_1_Br_3_*_n+_*_2_ (Type 2) are dominated by Br and Sn states. A sharp and strong peak (indicated by an arrow) appears for the DOS of Cs*_n_*Sn*_n+_*_1_Br_3_*_n+_*_2_ (*n* = 1), which indicates that the generation of an indirect bandgap is induced by Sn atoms. The intensity of the peak (indicated by an arrow) of Cs*_n_*Sn*_n+_*_1_Br_3_*_n+_*_2_ decreases gradually with layer numbers from 1 to 5, which means a transition from indirect to direct bandgap for Cs*_n_*Sn*_n+_*_1_Br_3_*_n+_*_2_. Therefore, according to the DOS in Figure 5, Cs*_n+_*_1_Sn*_n_*Br_3_*_n+_*_1_ is a direct bandgap and Cs*_n_*Sn*_n+_*_1_Br_3_*_n+_*_2_ is an indirect bandgap led by Sn atoms.

**Figure 6:**
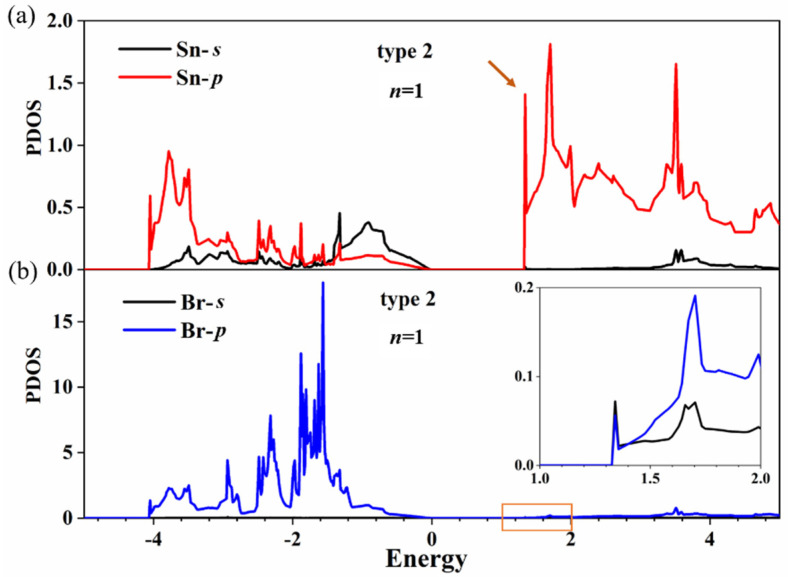
PDOS of Sn (**a**) and Br (**b**) atoms in the SnBr_2_-terminated surface for Cs*_n_*Sn*_n_*_+1_Br_3*n*+2_ (*n* = 1). The inset of (**b**) is an enlargement of the orange box.

Further efforts were made to separately calculate the partial density of states (PDOS) of Sn and Br atoms at the terminated surface in Figure 6 (the two atoms are indicated by arrows in Supplementary Figure S2). The results show that the conduction band bottom of Cs*_n_*Sn*_n+_*_1_Br_3_*_n+_*_2_ is mainly dominated by the 5*p*-Sn state. The peak of total DOS of Sn atoms in Figure 5b is 2.87, and the peak of PDOS of the Sn atom in Figure 6a is 1.41; there is about a twofold relationship between the size of the peak. It is also found that the contribution of the 4*p*-Br state to the conduction band bottom of Cs*_n_*Sn*_n+_*_1_Br_3_*_n+_*_2_ is negligible according to Figure 6b. Therefore, the 5*p-*Sn state at the terminated surface induced the generation of an indirect bandgap for Cs*_n_*Sn*_n+_*_1_Br_3_*_n+_*_2_. In addition, we calculated the orbital-projected band structures of Cs*_n_*Sn*_n+_*_1_Br_3_*_n+_*_2_ (*n* = 1) in Supplementary Figure S2a, which coincides completely with the calculated band structures in Figure 4a. This alignment indicates that the conduction band bottom of Cs*_n_*Sn*_n+_*_1_Br_3_*_n+_*_2_ is dominated by the 5*p*-Sn state, which agrees well with the PDOS calculations for Sn and Br atoms. 

A suitable bandgap energy and large absorption coefficient are important for photoelectric and photovoltaic devices. Therefore, the optical absorption of Cs*_n+_*_1_Sn*_n_*Br_3_*_n+_*_1_ and Cs*_n_*Sn*_n+_*_1_Br_3_*_n+_*_2_, with different layer numbers, have also been studied in this work. The optical absorption is generally calculated using the complex dielectric function, which is expressed as *ɛ*(ω) = *ɛ*_1_(ω) + i*ɛ*_2_(ω), where ω is the frequency of light, *ε*_1_ and *ε*_2_ are the real and imaginary parts of the dielectric function, respectively. *ε*_2_ is usually used to describe the light absorption behavior and its specific description is given by the following equation [57]:

(3) $$\varepsilon_{2}\left(\omega \right)=\frac{Ve^{2}}{2\pi \hbar m^{2}\omega^{2}}\int d^{3}k{\sum_{nn^{\prime }}\left|\langle kn|p{|kn\rangle }^{\prime }\right|}^{2}f\left(kn\right)*\left(1-f\left(kn^{\prime }\right)\right)\delta \left(E_{kn}-E_{kn^{\prime }}-\hbar \omega \right),$$

where *V* is unit volume, *e* represents electron charge, *m* is the electron rest mass, *p* is the momentum transition matrix, *ħ* is the reduced Planck Constant; *kn* and *kn′* are the wave functions of the conduction band and valence band, respectively. In order to rapidly distinguish these physical variables, we show them in the Supplementary Table S1. Moreover, by using the Kramers–Kronig relationship, the real part of the dielectric function is obtained as follows [58]:

(4) $$\varepsilon_{1}\left(\omega \right)=1+\frac{2}{\pi }P\int_{0}^{\infty }\frac{\varepsilon_{2}\left(\omega^{\prime }\right)\omega^{\prime }d\omega^{\prime }}{\omega^{\prime }{}^{2}-\omega^{2}},$$

where *P* is the principal value of the integral. The absorption coefficient is given as follows [59]:

(5) $$\alpha =2\omega {\left[\frac{{\left(\varepsilon_{1}^{2}\left(\omega \right)+\varepsilon_{2}^{2}\left(\omega \right)\right)}^{1/2}-\varepsilon_{1}\left(\omega \right)}{2}\right]}^{1/2},$$

**Figure 7:**
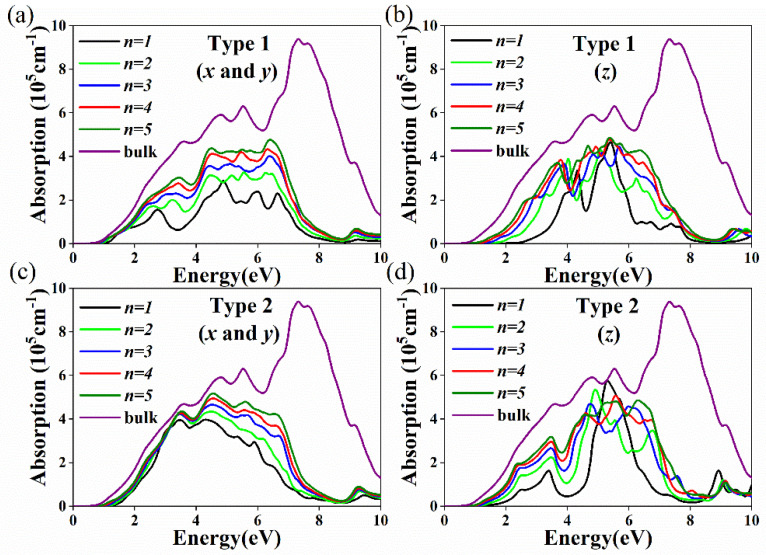
Calculated absorption coefficient for (**a**) Cs*_n_*_+1_Sn*_n_*Br_3*n*+1_ (*n* = 1–5) *x* and *y* directions; (**b**) Cs*_n_*_+1_Sn*_n_*Br_3*n*+1_ (*n* = 1–5) *z* directions; and (**c**) Cs*_n_*Sn*_n_*_+1_Br_3*n*+2_ (*n* = 1–5) *x* and *y* directions; (**d**) Cs*_n_*Sn*_n_*_+1_Br_3*n*+2_ (*n* = 1–5) *z* directions.

The absorption coefficient is a key parameter and is of great significance in photoelectric and photovoltaic applications. Figure 7 shows the absorption spectra of Cs*_n+_*_1_Sn*_n_*Br_3*n+*1_ and Cs*_n_*Sn*_n+_*_1_Br_3*n+*2_ with different layer numbers along the *x*, *y* (Figure 7a,c) and *z* (Figure 7b,d) directions. Both Cs*_n+_*_1_Sn*_n_*Br_3*n+*1_ and Cs*_n_*Sn*_n+_*_1_Br_3*n+*2_ have large light absorption coefficients in the visible and infrared regions. With the decrease in layer number, the absorption coefficients of both Cs*_n+_*_1_Sn*_n_*Br_3*n+*1_ and Cs*_n_*Sn*_n+_*_1_Br_3*n+*2_ have redshift. Different from that of bulk CsSnBr_3_, the light absorption of Cs*_n+_*_1_Sn*_n_*Br_3*n+*1_ and Cs*_n_*Sn*_n+_*_1_Br_3*n+*2_ show anisotropy. In the visible region, the absorption coefficients for Cs*_n_*Sn*_n+_*_1_Br_3*n+*2_ along the *x* and *y* directions are very close to and smaller than those for bulk CsSnBr_3_ (Figure 7c); the layer dependence is not strong. This behavior is related to the absorption of Sn and Br atoms in the terminated surface. Cs*_n_*Sn*_n+_*_1_Br_3*n+*2_ has a larger absorption coefficient than Cs*_n+_*_1_Sn*_n_*Br_3*n+*1_ along the *x* and *y* directions (Figure 7a,c). The SnBr-terminated surface model provides an ideal model for the design of 2D RP perovskites for photovoltaic and optoelectronic devices.

## 4. Conclusions

In conclusion, based on the cubic CsSnBr_3_, we designed two models in this work, including Cs*_n_*_+1_Sn*_n_*Br_3*n*+1_ with CsBr-termination and Cs*_n_*Sn*_n_*_+1_Br_3*n*+2_ with SnBr_2_-termination. Their bandgap energies, structural and optoelectronic properties of the two models were calculated using DFT. The calculated results indicated that the band structure of Cs*_n_*_+1_Sn*_n_*Br_3*n*+1_ is a direct bandgap. Additionally, the band structure of Cs*_n_*Sn*_n_*_+1_Br_3*n*+2_ can be altered from an indirect to direct bandgap with the increase in the layer numbers. With the variation of the layer number from 1 to 5, the bandgaps of Cs*_n_*_+1_Sn*_n_*Br_3*n*+1_ and Cs*_n_*Sn*_n_*_+1_Br_3*n*+2_ decreased from 1.209 to 0.797 eV and 1.310 to 1.013 eV, respectively. Furthermore, we calculated the DOS of Sn and Br atoms and the orbital-projected band structures of Cs*_n_*Sn*_n+_*_1_Br_3*n+*2_ (*n* = 1) in the terminated surface. It was found that the 5*p*-Sn state was responsible for the appearance of the indirect bandgap of Cs*_n_*Sn*_n+_*_1_Br_3*n+*2_. In addition, both Cs*_n+_*_1_Sn*_n_*Br_3*n+*1_ and Cs*_n_*Sn*_n+_*_1_Br_3*n+*2_ have as large of an absorption coefficient as bulk CsSnBr_3_ and show anisotropy. Nevertheless, Cs*_n_*Sn*_n+_*_1_Br_3*n+*2_ exhibits an insensitivity to the layer number along the *x* and *y* directions. The calculated results obtained in this work may provide new ideas for the design of photovoltaic devices.

## Figures and Tables

**Table 1:** Calculated relative displacements, *δ_i,i_*_+1_ (%, in unit of the calculated bulk CsSnBr_3_ lattice constant), and layers rumpling, *η_i_* (%), for Cs*_n+_*_1_Sn*_n_*Br_3*n+*1_ and Cs*_n_*Sn*_n+_*_1_Br_3*n+*2_ (*n* = 1–5).

Layer (*n*)			1	2	3	4	5
*δ_i,i+_*_1_ (%)	Type 1	Br_1_Sn_2_	0.6	−1.05	−1.56	−1.64	−1.71
		Sn_2_Br_3_		1.69	2.28	2.62	2.6
		Br_3_Sn_4_			0.004	−0.49	−0.69
		Sn_4_Br_5_				0.62	0.79
		Br_5_Sn_6_					0.02
	Type 2	Sn_1_Br_2_	−1.98	−2.68	−2.87	−2.9	−3
		Br_2_Sn_3_		0.24	1.14	1.52	1.69
		Sn_3_Br_4_			−0.74	−1.01	−1.13
		Br_4_Sn_5_				0.055	0.33
		Sn_5_Br_6_					−0.25
*η_i_* (%)	Type 1	Cs_1_Br_1_Cs_1_	−6.44	−8.28	−8.89	−8.78	−8.89
		Br_2_Sn_2_Br_2_	0	−1.78	−1.94	−1.89	−1.94
		Cs_3_Br_3_Cs_3_		0	−2.44	−3.11	−3.44
		Br_4_Sn_4_Br_4_			0	−0.17	−0.33
		Cs_5_Br_5_Cs_5_				0	−0.72
		Br_6_Sn_6_Br_6_					0
	Type 2	Br_1_Sn_1_Br_1_	−3.22	−3.28	−3.17	−2.94	−2.83
		Cs_2_Br_2_Cs_2_	0	−2.11	−2.83	−3.28	−3.67
		Br_3_Sn_3_Br_3_		0	−0.078	−1	−1
		Cs_4_Br_4_Cs_4_			0	−0.89	−1.28
		Br_5_Sn_5_Br_5_				0	−0.22
		Cs_6_Br_6_Cs_6_					0

**Table 2:** Calculated bandgap energies (in eV) of Cs*_n_*_+1_Sn*_n_*Br_3*n*+1_ and Cs*_n_*Sn*_n_*_+1_Br_3*n*+2_ (*n* = 1–5). The *d* and *ind* in parentheses represent direct and indirect bandgap, respectively.

Layer (*n*)	1	2	3	4	5	Bulk
Type 1	1.209 (*d*)	1.036 (*d*)	0.928 (*d*)	0.851 (*d*)	0.797 (*d*)	0.641
Type 2	1.310 (*ind*)	1.266 (*ind*)	1.198 (*ind*)	1.100 (*d*)	1.013 (*d*)	

## Data Availability

Data are contained within the article.

## Supplementary Materials

The following are available online at https://www.mdpi.com/article/10.3390/nano11082119/s1, Figure S1: band structure of Cs*_n_*Sn*_n+_*_1_Br_3*n+*2_ (*n* = 1) calculated by PBE (black lines) and HSE06 (red lines) DFT; Figure S2: (a) Orbital-projected band structures of Cs*_n_*Sn*_n+_*_1_Br_3*n+*2_ (*n* = 1). (b) Model of Cs*_n_*Sn*_n+_*_1_Br_3*n+*2_ (*n* = 1); Table S1. the variables used in this work, including variables’ names and physical meanings.
