# Supplementary material for: Structural and Optoelectronic Properties of Two-Dimensional Ruddlesden–Popper Hybrid Perovskite CsSnBr3
Source: Nanomaterials (Basel). 2021 Aug 20;11(8):2119. doi: 10.3390/nano11082119 (PMC8398644; doi:10.3390/nano11082119)
Supplement: Supplementary file 1 [file nanomaterials-11-02119-s001.zip › nanomaterials-1333765-supplementary.pdf]

Supplement Materials

Structural and Optoelectronic Properties of Two-Dimensional Ruddlesden–Popper Hybrid Perovskite $CsSnBr_3$

Guangbiao Xiang [1], Yanwen Wu [1], Yushuang Li [1], Chen Cheng [1,*], Jiancai Leng [2,*] and Hong Ma [1,*]

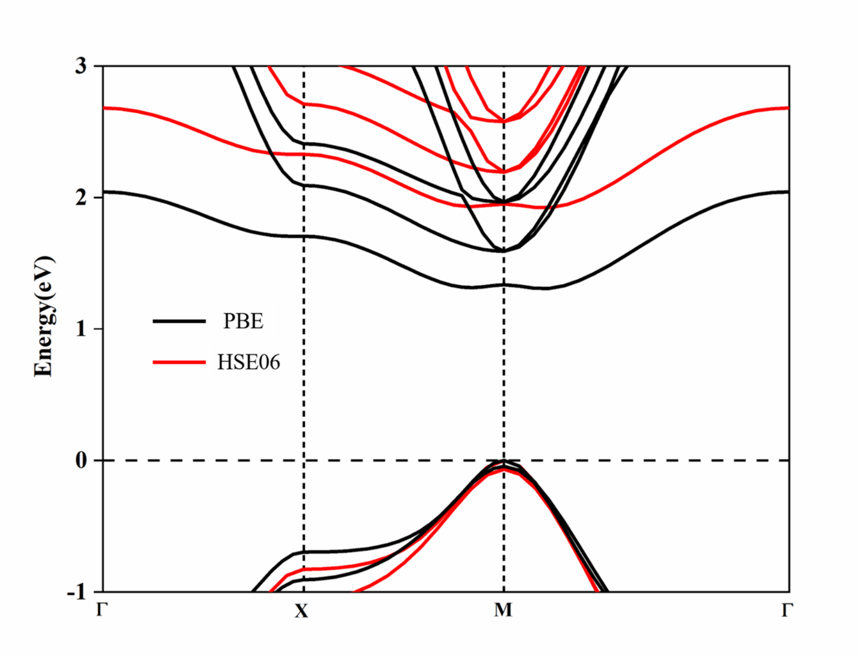

Figure S1. band structure of $Cs_nSn_{n+1}Br_{3n+2}$ (n=1) calculated by PBE (black lines) and HSE06 (red lines) DFT.

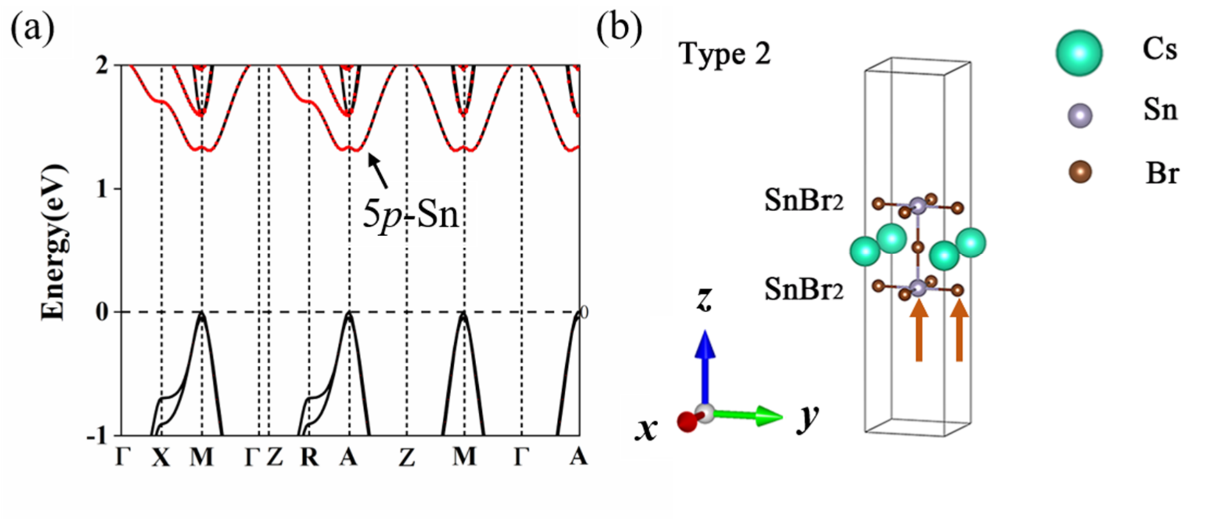

Figure S2. (a) Orbital-projected band structure of $Cs_nSn_{n+1}Br_{3n+2}$ (n = 1). (b) Model of $Cs_nSn_{n+1}Br_{3n+2}$ (n = 1). The arrow points to the Sn and Br atoms in $SnBr_2$-terminated surface.

Table S1. All variables use in this work, including variables name and physical meanings.

variables	physical meanings	variables	physical meanings
a_0	lattice constant	P	principal value of the integral
n	layers	p	momentum transition matrix
d	interplanar distance		dielectric function
δ	relative displacements	ω	frequency of light
$_i$	layers rumpling	α	absorption coefficient
θ	angle degree	$\hbar$	reduced Planck Constant
V	unit volume	e	electron charge
ε_1	real part of the dielectric function	ε_2	imaginary part of the dielectric function
kn	wave function of the conduction band	kn'	wave function of the valence band
